# Coughing, sneezing, and aching online: Twitter and the volume of influenza-like illness in a pediatric hospital

**DOI:** 10.1371/journal.pone.0182008

**Published:** 2017-07-28

**Authors:** David M. Hartley, Courtney M. Giannini, Stephanie Wilson, Ophir Frieder, Peter A. Margolis, Uma R. Kotagal, Denise L. White, Beverly L. Connelly, Derek S. Wheeler, Dawit G. Tadesse, Maurizio Macaluso

**Affiliations:** 1 James M. Anderson Center for Health Systems Excellence, Cincinnati Children's Hospital Medical Center, Cincinnati, Ohio, United States of America; 2 Medical Scientist Training Program, College of Medicine, University of Cincinnati, Cincinnati, Ohio, United States of America; 3 Department of Computer Science, Georgetown University, Washington DC, United States of America; 4 Division of Infectious Diseases, Cincinnati Children's Hospital Medical Center, Cincinnati, Ohio, United States of America; 5 Division of Critical Care Medicine, Cincinnati Children's Hospital Medical Center, Cincinnati, Ohio, United States of America; 6 Division of Biostatistics and Epidemiology, Cincinnati Children's Hospital Medical Center, Cincinnati, Ohio, United States of America; Hokkaido University Graduate School of Medicine, JAPAN

## Abstract

This study investigates the relation of the incidence of georeferenced tweets related to respiratory illness to the incidence of influenza-like illness (ILI) in the emergency department (ED) and urgent care clinics (UCCs) of a large pediatric hospital. We collected (1) tweets in English originating in our hospital’s primary service area between 11/1/2014 and 5/1/2015 and containing one or more specific terms related to respiratory illness and (2) the daily number of patients presenting to our hospital’s EDs and UCCs with ILI, as captured by ICD-9 codes. A Support Vector Machine classifier was applied to the set of tweets to remove those unlikely to be related to ILI. Time series of the pooled set of remaining tweets involving any term, of tweets involving individual terms, and of the ICD-9 data were constructed, and temporal cross-correlation between the social media and clinical data was computed. A statistically significant correlation (Spearman ρ = 0.23) between tweets involving the term *flu* and ED and UCC volume related to ILI 11 days in the future was observed. Tweets involving the terms *coughing* (Spearman ρ = 0.24) and *headache* (Spearman ρ = 0.19) individually were also significantly correlated to ILI-related clinical volume four and two days in the future, respectively. In the 2014–2015 cold and flu season, the incidence of local tweets containing the terms *flu*, *coughing*, and *headache* were early indicators of the incidence of ILI-related cases presenting to EDs and UCCs at our children’s hospital.

## Introduction

Social media represents a new and important source of data for healthcare researchers [1–7]. Of particular interest is the use of social media for community situational awareness to support local public health decision-making. While a recent review found few peer reviewed studies documenting the operational use of social media for infectious disease surveillance [8,9], analyses of different social media platforms demonstrate agreement with traditional clinic-based surveillance nationally and regionally [10,11] in the case of influenza-like illness (ILI). More recently, correlation between the incidence of georeferenced Twitter messages (“tweets”) containing words related to ILI and the clinical incidence of ILI as collected by city health departments in the United States was observed [12–15]. Such studies are important steps toward establishing Twitter as a proxy for ILI incidence collected by public health departments at the city-scale.

The degree to which the association of Twitter and ILI activity generalizes to clinical observation at specific hospitals, types of hospitals, and patient populations, however, is unclear and remains largely unexplored in the literature. The purpose of this study is to analyze potential correlation between the incidence of explicitly georeferenced tweets related to respiratory illness in the hospital catchment community to the incidence of ILI in emergency departments (EDs) and urgent care clinics (UCCs), as recorded in International Classification of Diseases, Ninth Revision (ICD-9) codes, in a large children’s hospital. While many hospitals see patients of all ages, including those of ages who use Twitter, children’s hospitals typically serve patients spanning the neonatal through young adult ages. Since Twitter’s services are directed at persons 13 years of age and older, an important demographic of children’s hospitals demographics may utilize Twitter. Moreover, a recent study suggests that 37% of adult Internet users between 18 and 29 years of age use Twitter, as do 25% of those between the ages of 30 and 49 [16]. The age range 18–49 includes the majority of ages of parents or guardians who bring children to pediatric hospitals for care as well as older patients presenting to children’s hospitals [17]. Therefore, Twitter likely is a social media platform relevant to pediatric health, though few studies address its relevance to pediatric infectious disease specifically.

## Methods

### Setting

Cincinnati Children’s Hospital is an academic primary through tertiary care institution serving patients throughout southwestern Ohio, northern Kentucky, and southeastern Indiana. Cincinnati Children’s Hospital has approximately 1.2 million patient encounters annually, of which approximately 100,000 are ED visits. The hospital maintains emergency rooms at two locations and urgent care clinics at five facilities in the Cincinnati, Ohio metropolitan area. Our hospital defines its primary service area according to the geographic boundaries of counties in the Ohio-Indiana-Kentucky tristate region. In this study, we approximated this region as a circle of 55-mile radius centered about Cincinnati.

### Data sources

Tweets containing the terms *nausea*, *nauseous*, *coughing*, *wheezing*, *asthma*, *sneezing*, *headache*, *achy*, *antibiotics*, and *flu* were collected from November 1, 2014—May 1, 2015 from within the hospital service area via the Twitter search application program interface (API) (https://dev.twitter.com/rest/public/search). The keywords *flu*, *headache*, and *coughing* derived from previous studies [14,15,18,19], while the others were chosen based on the symptoms of respiratory illness more generally. Data fields retained from the API queries included: *text*, *created*, *truncated*, *longitude*, and *latitude*. We utilized only tweets with explicit latitude and longitude coordinates in this study. The Twitter search API was queried nightly at midnight local time through the R programming language [20] employing the twitteR library [21]. The previous 24 hours of tweets were queried in each search so that only new tweets were returned in each query. Re-tweets, tweets containing the terms *ebola* and *game* (to decrease the number of irrelevant tweets and thus reduce the class imbalance problem for SVM [22]), and tweets containing URLs (which frequently denote news reports), were excluded from the queries.

For the same time period, data on the number of patients presenting to EDs and UCCs with ILI were queried from our hospital’s electronic health record (EHR) system. We developed a group of ICD-9 codes related to ILI in our patient population. Hospital infectious disease physicians selected ICD-9 codes from a list taken from the study of Marsden-Haug et al [23], which was done on a population including all ages, and added codes thought to be relevant based on clinical experience. Codes 465.9 (upper respiratory infection, acute, not otherwise specified), 487.1 (influenza with other respiratory manifestations), and 486 (pneumonia, organism unspecified), which were among the ICD-9 codes found to be most indicative of ILI in the Marsden-Haug et al study, were included in the present study. Also included were the following: 465 (acute upper respiratory infections of multiple or unspecified sites), 466 (acute bronchitis and bronchiolitis), 466.0 (bronchitis, acute), 466.11 (bronchiolitis, acute, due to RSV), 480 (viral pneumonia), 480.9 (pneumonia, viral, unspecified), 481 (pneumococcal pneumonia), 482 (other bacterial pneumonia), 482.9 (pneumonia, bacterial, unspecified), 483 (pneumonia due to other specified organism), 483.0 (mycoplasma pneumoniae), 485 (bronchopneumonia, organism unspecified), 487 (influenza with pneumonia), 490 (bronchitis, not specified as acute or chronic), 493 (asthma), 493.0 (extrinsic asthma), 493.1 (intrinsic asthma), 493.2 (chronic obstructive asthma), 518 (other diseases of lung), 518.5 (ARDS), 518.81 (respiratory failure, acute), and 519 (other diseases of respiratory system). The resulting data were stored as records including fields for visit time, date, and specific ICD-9 codes related to the visit.

### Data preparation

We manually classified half of the collected tweets to train a machine classifier. The classification rule applied was that if it was thought that the person tweeting or someone near them was suffering the condition in question (e.g., headache, coughing, asthma, aching, taking antibiotics, etc.) as a health problem potentially related to ILI, then a tweet was classified as relevant; otherwise, it was classified as irrelevant. The manual classification was based on a consensus of two researchers; *a priori* inter-rater reliability was assessed with Cohen’s Kappa (κ). We parsed the different terms (words or phrases) after cleaning the tweets. Cleaning (i.e., removal of stop words, punctuation, and numbers and conversion of all words to lower case) was accomplished using the R text mining package tm [24]. Search terms in the resulting tweets served as the variables for classification. We fit a Support Vector Machine (SVM) classifier using two-class classification using the package e1071 in R [25]. Standard SVM kernels were considered (linear, radial, polynomial and sigmoidal) and we adopted the kernel yielding the highest classifier accuracy based on 10-fold cross validation using the training data (50% of the entire twitter data set). Accuracy is defined as the number of true “relevant” and true “non-relevant” tweets divided by the number of “relevant” and “non-relevant” tweets. The resulting SVM classifier classified the remaining tweets.

### Statistics

For each type of data (i.e., tweets and ICD-9 codes) we constructed the daily incidence time series. In the case of Twitter, we constructed time series for both the sum of all tweets and the sum of tweets including individual keywords for the study area. We investigated the normality of the data corresponding to the resulting Twitter and ICD-9 time series through Shapiro-Wilk tests (test statistic: W) and applied transformations when needed to achieve normality. Augmented Dickey-Fuller tests were used to assess the stationarity of times series. The relationship between the time series was assessed using Spearman’s (ρ) rank-based correlation coefficient.

We removed autocorrelation in the time series [26] through pre-whitening. Following the standard procedure [27], we examined the autocorrelation function (ACF) and partial autocorrelation function to estimate an ARIMA(p,d,q) model to the Twitter series, where p is the order of the autoregressive model, d is the number of differences needed to achieve stationarity, and q is order of the moving average model. We filtered this series with the model to recover the white noise residual series, and then filtered the ICD-9 series with the same model. The filtered Twitter and ICD-9 series were then cross-correlated to estimate the correlation at different lags. Residuals from all ARIMA models were tested using Shapiro-Wilk tests and quantile-quantile (QQ) plots to confirm that they were normally distributed. Pre-whitening was carried out in the R programming language [20].

### Ethics statement

Tweets used in this study were collected from users who consented, via the Twitter terms and agreement, to make their tweets available publicly. No fields containing Twitter user identification were stored when collecting tweets from the search API. EHR data were de-identified prior to use in the study. The Cincinnati Children’s Hospital Medical Center Institutional Review Board determined that this study does not meet the criteria for human subjects research and therefore exempted the research from IRB approval.

## Results

**Fig 1:**
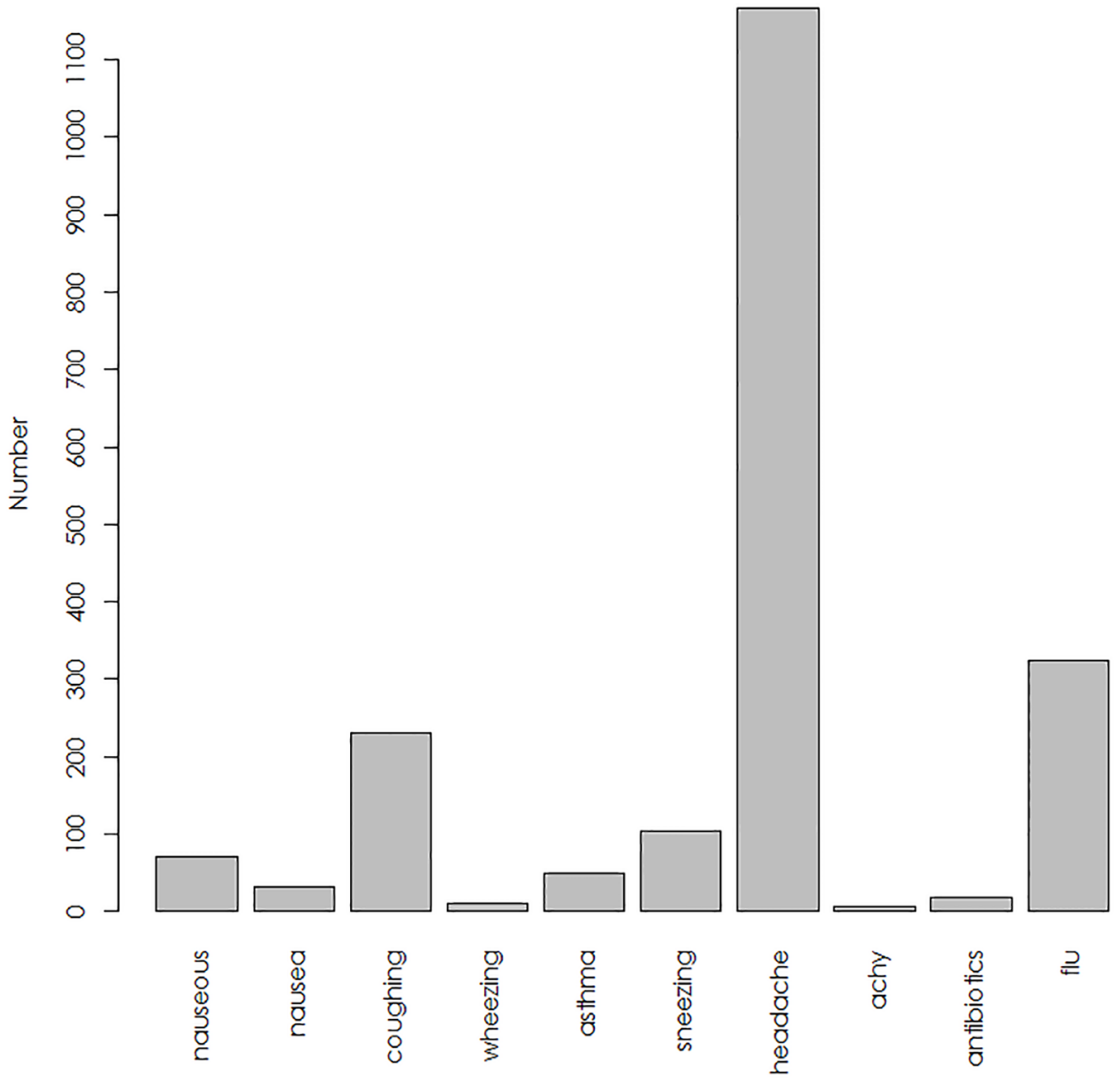
The frequency of occurrence of individual search terms in the 2057 tweets analyzed in this study.

We collected a total of 2737 tweets. Overall, *headache* was the most common keyword (occurring in 53.8% of tweets), followed by *flu* (occurring in 19.2% of tweets) and *coughing* (occurring in 10.8% of tweets); other terms occurred much less commonly. The *a priori* inter-rater reliability identifying relevant versus irrelevant tweets was high (κ = 0.82). Based on 10-fold cross validation, we found that an SVM classifier based on a linear kernel performed better than classifiers utilizing radial, polynomial or sigmoidal kernels, and yielded an average accuracy of 78%. Applying the classifier to the remaining tweets resulted in 2057 tweets retained for analysis. As shown in Fig 1, a similar distribution of terms was observed in the machine-classified data set: *headache* occurred in 57.7% of tweets, *flu* occurred in 16.3% of tweets, and *coughing* occurred in 11.4% of tweets while other terms occurred much less commonly.

**Fig 2:**
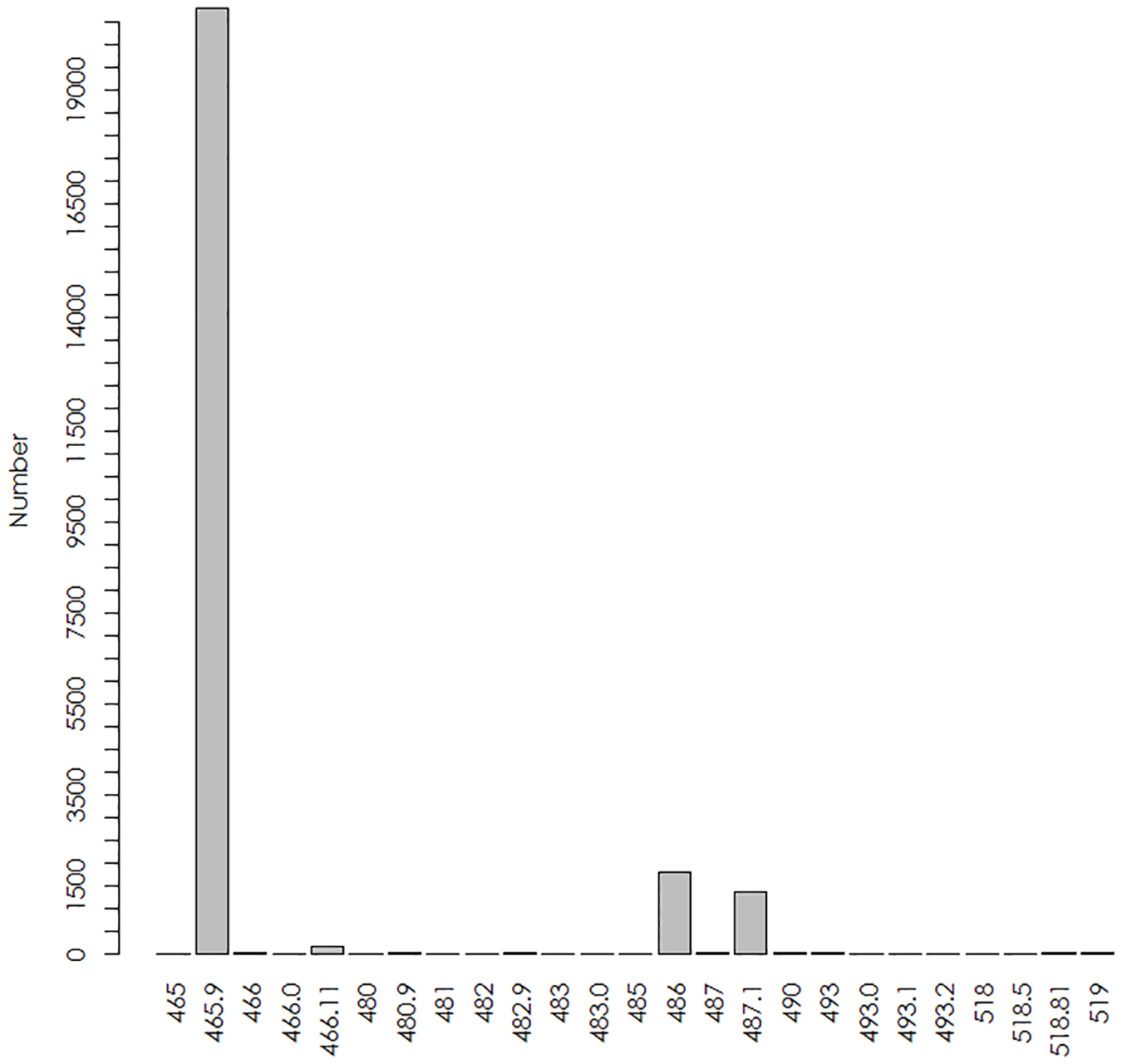
The frequency of occurrence of individual ICD-9 codes in the EHR records analyzed in this study.

The EHR query yielded 12081 cases seen in hospital EDs and UCCs, in which ICD-9 codes occurred with the frequencies depicted in Fig 2. ICD-9 code 465.9 (“upper respiratory infection, acute, NOS”) was by far the most common admit and discharge code, accounting for 85.9% of all codes collected, followed by codes 486 (“pneumonia, organism unspecified”, 7.4%) and 487.1 (“influenza w/other respiratory manifestations”, 5.6%); other codes occurred with negligible frequency.

**Fig 3:**
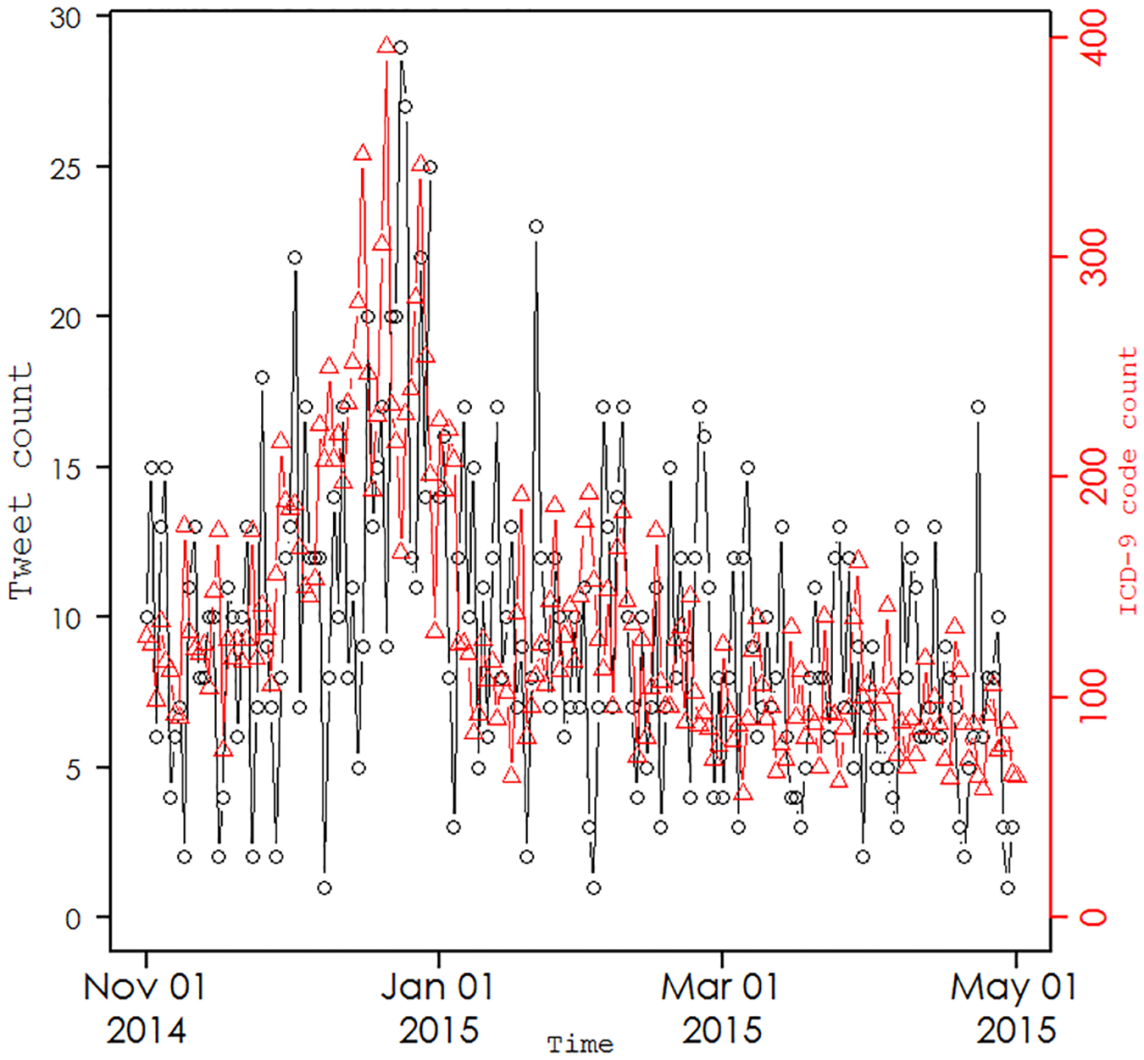
The daily incidence of the sum of tweets including the terms *flu*, *coughing*, and *headache* (black open circles) in the hospital catchment area and the daily volume of hospital ED and UCC ICD-9 codes (red triangles) related to ILI.

Based on their prominence relative to other terms, we considered only tweets containing the terms *flu*, *coughing*, and *headache* (dataset in S1 File). Fig 3 depicts the daily incidence of the sum of tweets including the terms *flu*, *coughing*, and *headache* (black open circles) in the hospital catchment area and the daily volume of hospital ED and UCC ICD-9 codes (red triangles) related to ILI. The Shapiro-Wilk test applied to the daily incidence of the sum of tweets including these three keywords (W = 0.95, p<0.001) and separately to daily volume of hospital ED and UCC ICD-9 codes related to ILI (W = 0.87, p<0.001) indicated that neither are likely to be normally distributed. Shapiro-Wilk tests were also applied to the time series of individual keywords; in each case, the test led to rejection of the null hypothesis that the data were from a normal distribution. Autocorrelation was observed in the ACFs of the time series of individual keywords.

We transformed the time series of tweets using square root function in order to achieve normality of all the pre-whitened time series of tweets. We used Dickey-Fuller tests for each pre-whitened series and each time found stationarity. We examined the ACF of each of the pre-whitened series and the residuals and observed no significant autocorrelation. We found that ARIMA models of order (p = 1, d = 1, q = 2) for *flu*, (p = 3, d = 2, q = 0) for *coughing*, and (p = 0, d = 0, q = 1) for *headache* best fit the data. Cross-correlation of the filtered time series identified statistically significant correlation at lags of -11 days (ρ = 0.23), -4 days (ρ = 0.24), and -2 days (ρ = 0.19) for *flu*, *coughing*, and *headache*, respectively, and the ICD-9 time series. We also observed autocorrelation in the time series of the three pooled keywords from the autocorrelation and partial autocorrelation functions. Applying the pre-whitening procedure, we found that an ARIMA model of order (p = 0, d = 0, q = 2) best fit the data and this was used to generate the white noise residual series. Cross-correlation of this filtered time series produced statistically significant correlation at a lag of -2 days (ρ = 0.30) between the pooled tweets containing *flu* or *coughing* or and *headache* and the ICD-9 time series. When Bonferroni correction is made to control for multiple comparison, the resulting 95% confidence interval of the cross-correlation functions is [-0.17, 0.17], so that all correlations are statistically significant.

## Discussion

This study examined the temporal relation between tweets involving the words *flu*, *coughing*, and *headache* and the volume of patients presenting to EDs and UCCs with ILI at a children’s hospital during a large part of the 2014–2015 cold and flu season. We found that tweets involving the word *flu* were predictive of ED and UCC ILI volume at a lead time of 11 days. Tweets involving the words *coughing* and *headache* were less strongly correlated with ILI volume at lead times of four and two days, respectively. The pooled time series of tweets involving *flu*, *coughing*, or *headache* was also found to be correlated with, and predictive of, ILI-related ED and UCC volume.

Other investigators recently also noted correlation at the city level between trends in tweets involving the term *flu* and trends in ILI in the general population [14,18], though we believe this study documents the first observation of the association of the incidence of tweeting about ILI and ILI as observed at a children’s hospital. Moreover, the observation of a predictive lag between such tweets and ILI suggests that it may be possible to forecast increases in pediatric ED and UCC volume related to ILI, at modest lead times, by collecting and classifying tweets involving the word *flu* originating from our hospital’s catchment region.

The US Centers for Disease Control and Prevention observes that influenza activity in the United States typically begins to increase in October and November [28]. In the 2014–2015 season, CDC reported that ILI activity first exceeded the baseline during the week ending November 22 [29]. Therefore, while our study began the first week of November and thus did not capture the entire ILI season, omission of the month of October in our data should not significantly change the results of the study.

Consistent with numerous previous studies [14,15,18,19,30], we opted to utilize an *a priori* selection of keywords representing symptoms associated with ILI for Twitter queries. We found that, out of several keywords considered, *flu*, *coughing*, and *headache* appeared most commonly in the tweets collected, further strengthening the previously-established validity of these keywords. We elected to include only tweets that include latitude and longitude coordinates explicitly and not to utilize enhanced geo-location approaches such as CARMEN [31] or TwoFishes (http://twofishes.net/). Such approaches typically exploit, in addition to latitude-longitude coordinates attached to tweets (either directly or via place tagging, if the user allows), information in user profiles, or potentially language in the body of tweets. While limiting the potential data available for the study to the few percent of all tweets estimated to contain latitude and longitude coordinates [32], utilizing tweets containing explicit latitude-longitude coordinates garnered from GPS avoids ambiguity in location that user profile information, for example, introduces at the geographic scales of interest in this study. Dredze and coworkers suggest that such approaches are accurate to within 50 miles about 60 percent of the time [31].

The approach used in this study has several limitations. First, we do not know the degree to which tweets mined from the search API are representative of all tweets, and we are unaware of published studies estimating the size or randomness of API search results relative to the entire Twitter “fire hose”. Second, while we employed machine learning methods to remove irrelevant tweets (i.e., tweets unrelated to respiratory illness, such as, e.g., “His voice gives me a headache, LOL”) [33,34], the accuracy of the classifier could likely be improved if additional data were available to better inform the training. Third, because sample size would be insufficient, we did not disaggregate the ICD-9 time series by clinic or location. Nor did we investigate possible day-of-week effects (i.e., the possibility that people may be predisposed to tweet about ILI on certain days of the week relative to others [35]), though we are not aware of such effects from previous studies. Fourth, these keywords, while biologically inspired and consistent with those used in other studies, may have not been optimal [14,15,18,19]. Similarly, while we filtered out tweets including the words “game” and “ebola” in order to decrease the collection of irrelevant tweets, these terms are not necessarily generalizable to future years.

Study results suggest that changes in the incidence of Twitter messages originating near Cincinnati and containing the words *flu*, *coughing*, and *headache* portend changes in ED and UCC volume at the lags calculated. It is possible that this approach may be of highest value at the beginning of the cold and flu season, when Twitter is relatively quiescent in terms of messages related to ILI and before users may become sensitized to tweeting about symptoms. While this study examined the association between tweets including words related to ILI and pediatric ILI observed in clinical settings, it is also of interest to understand how ILI-related tweets vary in terms of age, gender, and other demographic factors. While doing so is difficult [36,37], applying such tools in future efforts may result in a rich set of factors to study. Lastly, it may be possible to improve the correlation through smoothing of the data collected (e.g., moving average schemes). Because our study has not included such approaches, the results of our analysis are likely to be biased toward the null. Employing methods such as those described in this study may strengthen the association between local tweets and pediatric ILI observed at our children’s hospital, support early warning of increases in ILI incidence, and lead to deeper insight into the relationship of social media and pediatric infectious disease at the community level.

## Supporting information

S1 File Excel file of the time series of tweet counts before classification (tab entitled “Tweet ts before classification”), tweet counts after classification (tab entitled “Tweet ts after classification”), and ICD-9 counts (tab entitled “ICD-9 code time series”). (XLS)
